# A systematic review, meta-analysis, and network analysis of diagnostic microRNAs in glaucoma

**DOI:** 10.1186/s40001-023-01093-8

**Published:** 2023-03-27

**Authors:** Masoud Rezaei, Mahsa Faramarzpour, Parnian Shobeiri, Homa Seyedmirzaei, Mohammad Sharifi Sarasyabi, Shahriar Dabiri

**Affiliations:** 1 Research Center for Hydatid Disease in Iran, Kerman University of Medical Sciences, Kerman, Iran; 2 Pathology and Stem Cell Research Center, Department of Pathology, Afzalipour Faculty of Medicine, Kerman University of Medical Sciences, Kerman, Iran; 3 School of Medicine, Tehran University of Medical Sciences, Tehran, Iran; 4 Universal Scientific Education and Research Network (USERN), Tehran, Iran; 5 Endocrinology and Metabolism Population Sciences Institute, Non–Communicable Diseases Research Center, Tehran University of Medical Sciences, Tehran, Iran; 6 Afzalipour Faculty of Medicine, Kerman University of Medical Sciences, Kerman, Iran; 7 Department of Radiology, Afzalipour Faculty of Medicine, Kerman University of Medical Sciences, Kerman, Iran

**Keywords:** Glaucoma, MicroRNA, Systematic review, Meta-analysis, Network analysis

## Abstract

Glaucoma is a chronic neurodegenerative process of the optic nerve that is the leading cause of blindness worldwide, and early diagnosis of the disease could greatly affect patients’ prognoses. The pathophysiology of glaucoma is complicated by a combination of genetic and epigenetic factors. Deciphering the early diagnostic biomarkers in glaucoma could attenuate the disease's global burden and help us understand the exact mechanisms involved in glaucoma. The microRNAs are members of a larger family of non-coding RNAs that play an essential role in the epigenetic basis of glaucoma. A systematic study and meta-analysis of diagnostic microRNAs in glaucoma, jointly with network analysis of target genes, were carried out on published papers assessing differentially expressed microRNAs in human subjects. In total, 321 articles were found, and, after screening, six studies were eligible for further analysis. 52 differentially expressed microRNAs were found, of which 28 and 24 were up-regulated and down-regulated, respectively. Only 12 microRNAs were qualified for meta-analysis, with overall sensitivity and specificity of 80% and 74%, respectively. Then, using network analysis, it became apparent that the VEGF-A, AKT1, CXCL12, and HRAS genes were the most important targets for the microRNAs. Perturbations in WNT signaling, protein transport, and extracellular matrix organization pathways were discovered to be important in the etiology of glaucoma using the community detection approach. This study tries to uncover the promising microRNAs and their target genes that govern the epigenetics of glaucoma.

**Supplementary Information:**

The online version contains supplementary material available at 10.1186/s40001-023-01093-8.

## Introduction

Glaucoma is a chronic neurodegenerative process within the optic nerve that is the leading cause of blindness, affecting more than 70 million people worldwide [1, 2]. The imperceptible and gradual nature of the disease leads to delayed diagnosis and irreversible neuropathy. Clinical diagnosis of glaucoma is relatively straightforward, but the delayed diagnosis puts a stop to the timely intervention; histopathologic changes are way before clinical changes in the optic disc and visual function [3]. The pathophysiology of glaucoma remains largely unknown. Our knowledge is limited except for several well-known risk factors, such as a strong family history of glaucoma, increased intraocular pressure, advanced age, corticosteroid use, and the black race [4]. Although increased intraocular pressure (IOP) is the most common and the only modifiable risk factor for glaucoma, in about thirty percent of patients with glaucoma, the IOP is within the normal range, which implies that there must be other contributing factors in the development of optic neuropathy [4, 5]. As we can infer from the risk factors indicated, there must be a combination of genetic and environmental interactions in the pathogenesis of glaucoma. There are various hypotheses for the pathophysiology of glaucoma that have yet to be completely explored, including neurotrophic factor deficiency, excitotoxicity, ischemia, oxidative stress, and axonal transport failure [6].

Epigenetics is the study of how environmental circumstances influence our genetics, and it should be acknowledged as an important component of glaucoma pathogenesis and development [7]. One of the key arms of the epigenetic regulatory effect is microRNAs (also called miRNAs). MicroRNAs are members of a larger family of non-coding RNAs, and the average length of microRNAs is 22 nucleotides [8].

MicroRNAs have garnered tremendous attention over the past few years due to the large number of processes they govern, such as cell growth and death. Basically, microRNAs interfere with messenger RNA translation, and in this way, they regulate about a third of human protein-coding genes [8]. Emerging studies suggests that the microRNAs could play a dual role in glaucoma. Protective microRNAs such as micro-RNA-483-3p decrease extracellular matrix fibrosis in response to stress. On the other hand, disease-promoting microRNAs such as microRNA-100 have been shown to reduce nerve growth [7]. A growing body of research has focused extensively on utilizing microRNAs in establishing the diagnosis and prognosis of chronic diseases such as glaucoma [9]. However, the accuracy and validity of these microRNAs in establishing the diagnosis of glaucoma are still under debate.

Identifying the most important evidence-based microRNAs can help us in the early diagnosis of the disease, in its prevention, and in the design of drugs that change the nature of the disease. Our study is nevertheless a valuable contribution to the field as it suggests a systematic review, meta-analysis, and genetic network study of the most critical microRNAs orchestrating the development of glaucoma.

## Materials and methods

### Databases and search strategy

PubMed, Web of Science, EMBASE, OVID, and Scopus databases were utilized, and papers from inception to December 30 2021 with the combination of the following search terms, were extracted: human, case or patient, control, microRNA, glaucoma, intraocular pressure, or hypertension. The full search strategy was shown in the method of literature search part.

### Method of literature search

("human" OR "humans" OR "patient" OR "patients" OR "control" OR "controls" OR "group" OR "groups") AND ("microrna" OR "micro RNA" OR "micrornas" OR "mirs" OR "microRNA") AND ("glaucoma" OR "intraocular hypertension" OR "intraocular pressure" OR "ocular hypertension").

### Inclusion and exclusion criteria

Articles are eligible for inclusion if they fulfill the following criteria: [1] original articles that are designed as a case–control study to assess the microRNA expression profiling in glaucoma patients; [2] the study must provide a source of samples obtained from the case and control groups [3]. MicroRNA profiling must be accomplished using one of the qPCR, microarray, or next-generation sequencing methods, and [4] the cut-off criteria for detection of differentially expressed microRNA must be reported. Review articles, studies not published in English, and animal and cell line studies were excluded.

### Data extraction

Two independent researchers (HS and MSS) screened, evaluated, and extracted the data to minimize the risk of selection bias in the study. In cases where two researchers disagreed on the inclusion or exclusion of an article, the third researcher (MR) made the definitive decision.

### Quality assessment

The quality of the included studies was assessed using the Quality Assessment of Diagnostic Accuracy Studies (QUADAS-2) criteria [10]. This method uses seven questions to evaluate the diagnostic accuracy studies in four key domains: patient selection, index test, reference standard, flow, and time. The seven questions are listed as follows: (1) Could the selection of patients have introduced bias? (2) Are there concerns that the included patients and setting do not match the review question? (3) Could the conduct or interpretation of the index test have introduced bias? (4) Are there concerns that the index test, its conduct, or its interpretation differ from the review question? (5) Could the reference standard, its conduct, or its interpretation have introduced bias? (6) Are there concerns that the target condition, as defined by the reference standard, does not match the question? (7) Could the patient flow have introduced bias? The first two address the assessment of patient selection; the next two address the index test; questions five and six address the reference standard; and the last one addresses the flow and timing.

### Statistical analysis

The analysis was conducted using receiver operator characteristic (ROC) curves to determine the accuracy of the discovered microRNAs in diagnosing glaucoma, as measured by the area under the curve (AUC) value and sensitivity and specificity, if available. The meta-analysis comprised studies that determined the sensitivity and specificity of particular microRNAs in diagnosing glaucoma. A random-effects model (DerSimonian–Laird technique) was utilized to account for the expected heterogeneity of investigations. All plots were constructed using STATA version 17 (StataCorp LP, College, Station, TX, USA). Also, all analyses were performed using STATA 17.0 software. The heterogeneity of included studies was assessed using I2 and χ2 statistics and was judged to be significant if I2 was > 50% or the *p-value* < 0.05. Where more than two studies of the same sample type were available, a subgroup analysis has been performed.

### Network analysis

#### microRNA target identification

The MirTarBase database was used to find the microRNA targets. The MirTarBase is one of the most comprehensive databases that contains experimentally validated microRNA targets [11]. Targets discovered using at least one of the robust evidence methods, namely the Reporter Assay, Western Blot, or qPCR for the searched microRNAs, were included in the subsequent analyses.

#### Protein–protein interaction (PPI) network construction and hub genes identification

The STRING database was used to construct the PPI network. For PPI network construction, a minimum interaction score of at least 0.7 (high confidence) was needed. Only interactions between the given genes were allowed, and additional interactions were not considered in the final network. The non-interacting genes were removed. To find the most critical genes in the network (i.e., hub genes), we used a Cytoscape plugin, CytoHubba (version 0.1), which is able to find the essential genes. The top ten genes with the highest maximal clique centrality were considered the hub genes. Maximal clique centrality (MCC) is the largest subset of a network in which every two nodes are connected by a vertex. It has been demonstrated that the MCC score is an efficacious topologic metric for finding hub nodes.

#### Community detection and prioritization

Communities (also called clusters) are densely connected components in a network with a distinctive function. To find the algorithm with the highest level of modularity, we utilized a Friedman ranking statistic filter approach based on modularity. In other words, the algorithm with the highest level of modularity ranks first, along with the statistical difference between algorithms. Several different algorithms (walk-trap, Markov clustering, surprise, and spectral algorithms) have been used in this benchmark. The CDlib Python library was used to implement the community detection algorithms and ranking process [12].

After finding the most appropriate algorithm for community detection, we will apply a community prioritizing algorithm called CRANK to identify the most promising ones for future experimentation. The gene set enrichment analysis will be applied to each community using the Enrichr server [13]. This approach helps us guide future studies cost-effectively and prevent time-consuming research on drug discovery and repurposing.

## Results

### Data acquisition and characteristics of the included studies

**Fig. 1:**
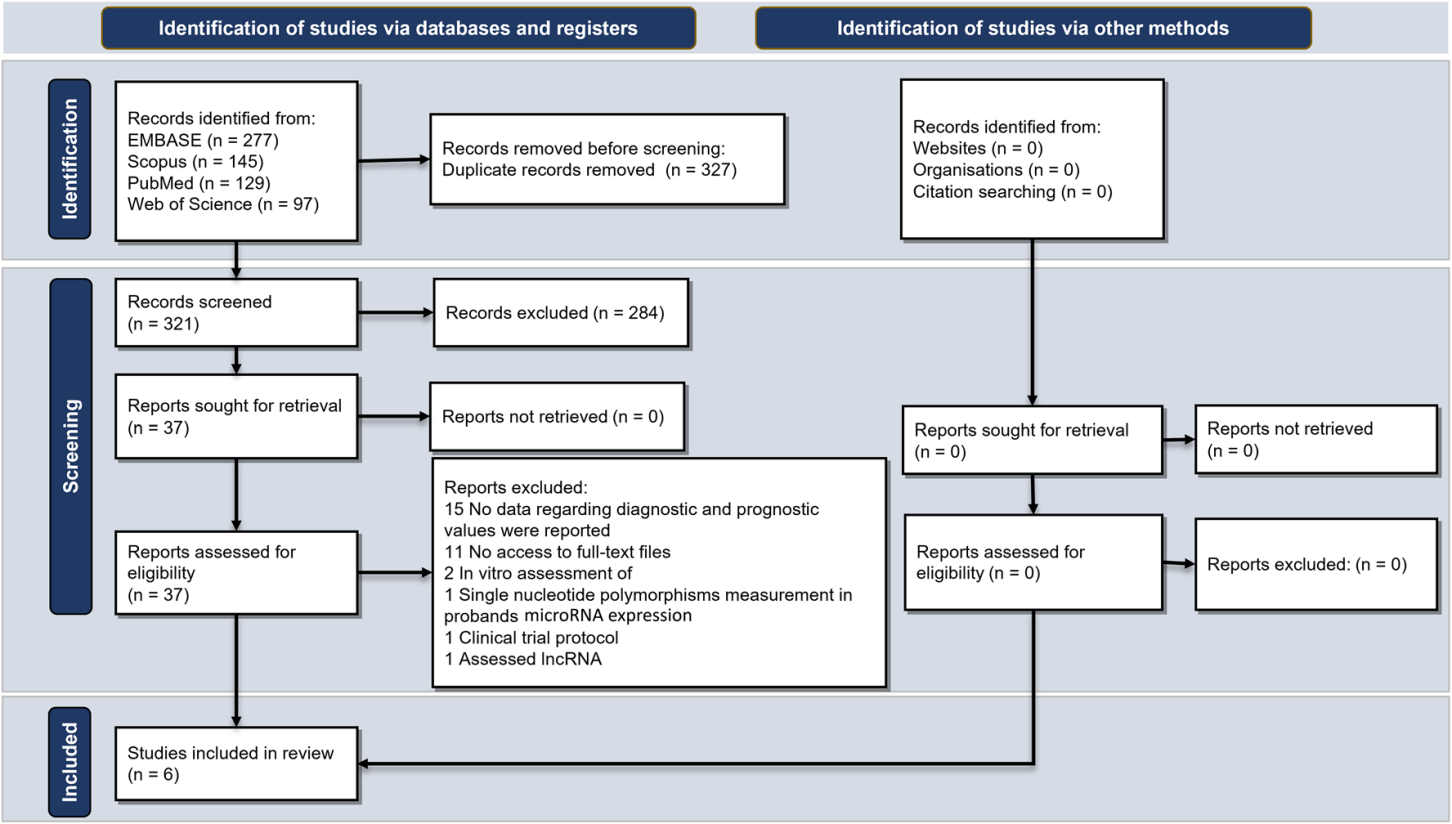
PRISMA flowchart

**Fig. 2:**
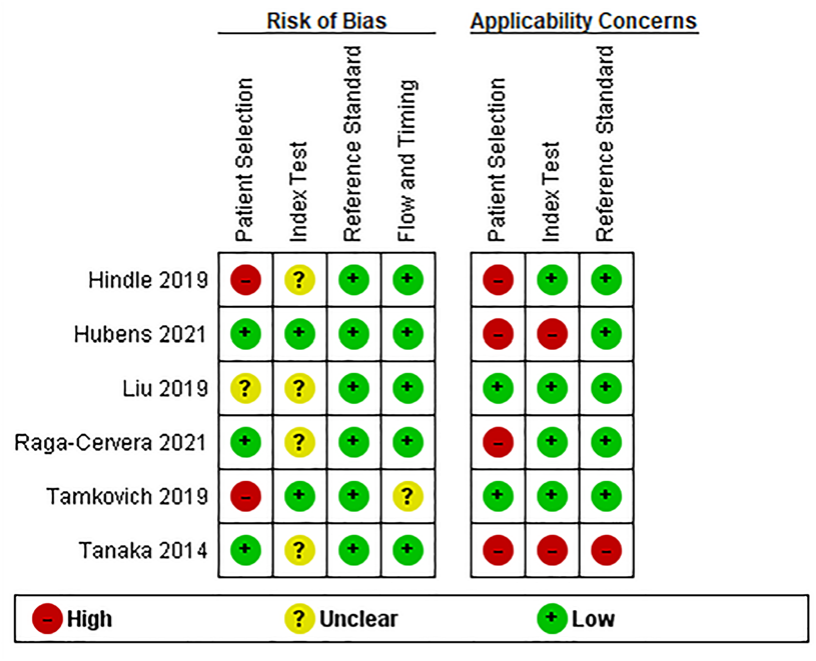
Summary of bias risk assessment results for QUADAS-2

**Fig. 3:**
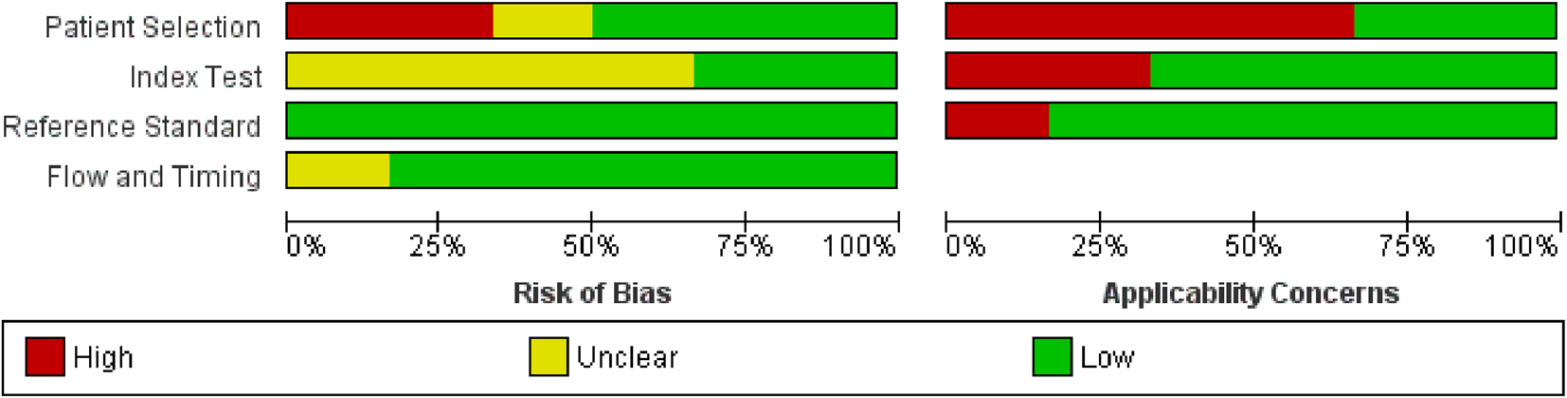
Quality of included studies according to QUADAS-2

The primary search and study selection procedure is briefly demonstrated as a diagram, famously known as Preferred Reporting Items for Systematic Reviews and Meta-Analyses (PRISMA), in Fig. 1 [14]. As shown, our search strategy retrieved a total of 648 studies. After removing 327 duplicates, 321 studies remained for the title/abstract screening. Respectively, 284 irrelevant studies were omitted, and out of the remaining 37 studies, 6 met the inclusion criteria and entered our study [15–20]. These case–control studies measured the microRNA levels in glaucoma patients and healthy people; two of them analyzed the aqueous humor [16, 20], two assessed tears [18, 19], and the other two investigated serum and plasma samples [15, 17]. Table 1 displays the extracted data from the included studies. While one study included only female participants [15], the rest assessed both females and males. Moreover, real-time polymerase chain reaction (RT-PCR), next-generation sequencing, and microarray systems were administered. The risk of bias analysis of the articles based on the QUADAS-2 checklist is summarized in Figs. 2 and 3.

**Table 1:** Differentially expressed microRNAs in all studies along with fold changes and study characteristics

	Author, year	Country	Sample	Sample size	Detection method	Glaucoma patients				Controls				Differentially expressed miRNA	Fold change/relative expression
						NO	Type	Mean age (± SD)	F/M	NO	Type	Mean age (± SD)	F/M		
1	Hindle et al.,2019	United States	Plasma	28	RT-PCR	17	POAG	74.67 (± 6.04)	17/0	11	Cataract, anatomical narrow angle	73 (± 4.74)	11/0	637	2.53 Up-regulated
2	Hindle et al.,2019	United States	Plasma	28	RT-PCR	17	POAG	74.67 (± 6.04)	17/0	11	Cataract, anatomical narrow angle	73 (± 4.74)	11/0	1306-5p	1.34 Up-regulated
3	Hindle et al.,2019	United States	Plasma	28	RT-PCR	17	POAG	74.67 (± 6.04)	17/0	11	Cataract, anatomical narrow angle	73 (± 4.74)	11/0	3159	2.31 Up-regulated
4	Hubens et al.,2021	The Netherlands	Aqueous humor	19	Next-generation sequencing	9	POAG	75.33 (± 4.98)	5/4	10	Cataract	75.8 (± 10.62)	3/7	30a-3p	Up-regulated
5	Hubens et al.,2021	The Netherlands	Aqueous humor	19	Next-generation sequencing	9	POAG	75.33 (± 4.98)	5/4	10	Cataract	75.8 (± 10.62)	3/7	143-3p	Up-regulated
6	Hubens et al.,2021	The Netherlands	Aqueous humor	19	Next-generation sequencing	9	POAG	75.33 (± 4.98)	5/4	10	Cataract	75.8 (± 10.62)	3/7	221-3p	Up-regulated
7	Hubens et al.,2021	The Netherlands	Aqueous humor	19	Next-generation sequencing	9	POAG	75.33 (± 4.98)	5/4	10	Cataract	75.8 (± 10.62)	3/7	211-5p	Up-regulated
8	Hubens et al.,2021	The Netherlands	Aqueous humor	19	Next-generation sequencing	9	POAG	75.33 (± 4.98)	5/4	10	Cataract	75.8 (± 10.62)	3/7	486-5p	Down-regulated
9	Hubens et al.,2021	The Netherlands	Aqueous humor	19	Next-generation sequencing	9	POAG	75.33 (± 4.98)	5/4	10	Cataract	75.8 (± 10.62)	3/7	451a	Down-regulated
10	Hubens et al.,2021	The Netherlands	Aqueous humor	19	Next-generation sequencing	9	POAG	75.33 (± 4.98)	5/4	10	cataract	75.8 (± 10.62)	3/7	92a-3p	Down-regulated
11	Liu et al.,2019	China	Serum	52	qRT-PCR	26	POAG	39.8 (± 12.1)	6/20	26	Healthy	39.6 (± 13.7)	8/18	210-3p (screening)	1.05 ± 0.08 fold in control 2.52 ± 0.55 fold in POAG Up-regulated
12	Liu et al.,2019	China	Serum	66	qRT-PCR	33	POAG	41.2 (± 12.7)	8/25	33	Healthy	42.8 (± 11.1)	7/26	210-3p (validation)	1.22 ± 0.15 fold in control 2.54 ± 0.39 fold in POAG Up-regulated
13	Liu et al.,2019	China	Serum	52	qRT-PCR	26	POAG	39.8 (± 12.1)	6/20	26	Healthy	39.6 (± 13.7)	8/18	885-5p (screening)	1.22 ± 0.18 fold in control 1.44 ± 0.32 fold in POAG Up-regulated
14	Raga-Cervera et al.,2021	Spain	Tears	42	Next-generation sequencing	20	POAG	64.5 (± 1.4)	52.4/47.6	22	Ocular hypertension without neurodegeneration signs (OHT)	61.1 (± 2.4)	66.7/33.3	26b-5p	0.855 Up-regulated
15	Raga-Cervera et al.,2021	Spain	Tears	42	Next-generation sequencing	20	POAG	64.5 (± 1.4)	52.4/47.6	22	Ocular hypertension without neurodegeneration signs (OHT)	61.1 (± 2.4)	66.7/33.3	27a-3p	0.774 Up-regulated
16	Raga-Cervera et al.,2021	Spain	Tears	42	Next-generation sequencing	20	POAG	64.5 (± 1.4)	52.4/47.6	22	Ocular hypertension without neurodegeneration signs (OHT)	61.1 (± 2.4)	66.7/33.3	152-3p	0.753 Up-regulated
17	Raga-Cervera et al.,2021	Spain	Tears	42	Next-generation sequencing	20	POAG	64.5 (± 1.4)	52.4/47.6	22	Ocular hypertension without neurodegeneration signs (OHT)	61.1 (± 2.4)	66.7/33.3	30e-5p	0.901 Up-regulated
18	Raga-Cervera et al.,2021	Spain	Tears	42	Next-generation sequencing	20	POAG	64.5 (± 1.4)	52.4/47.6	22	Ocular hypertension without neurodegeneration signs (OHT)	61.1 (± 2.4)	66.7/33.3	125b-2-5p	0.529 Up-regulated
19	Raga-Cervera et al.,2021	Spain	Tears	42	Next-generation sequencing	20	POAG	64.5 (± 1.4)	52.4/47.6	22	Ocular hypertension without neurodegeneration signs (OHT)	61.1 (± 2.4)	66.7/33.3	224-5p	0.745 Up-regulated
20	Raga-Cervera et al.,2021	Spain	Tears	42	Next-generation sequencing	20	POAG	64.5 (± 1.4)	52.4/47.6	22	Ocular hypertension without neurodegeneration signs (OHT)	61.1 (± 2.4)	66.7/33.3	151a-3p	− 0.417 Down-regulated
21	Raga-Cervera et al.,2021	Spain	Tears	42	Next-generation sequencing	20	POAG	64.5 (± 1.4)	52.4/47.6	22	Ocular hypertension without neurodegeneration signs (OHT)	61.1 (± 2.4)	66.7/33.3	1307-3p	− 1 Down-regulated
22	Tamkovich et al.,2019	Russia	Tears	62	RT-PCR	33	POAG	52–81 Y/O median 65	22/11	29	Healthy persons	44–79 Y/O Median 63		146-b	32.70 ± 0.67 in HPs 34.03 ± 1.17 in POAG Up-regulated
23	Tamkovich et al.,2019	Russia	Tears	62	RT-PCR	33	POAG	52–81 Y/O median 65	22/11	29	Healthy persons	44–79 Y/O Median 63		126	33.71 ± 0.54 in HPs 34.48 ± 0.70 in POAG Up-regulated
24	Tamkovich et al.,2019	Russia	Tears	62	RT-PCR	33	POAG	52–81 Y/O median 65	22/11	29	Healthy persons	44–79 Y/O Median 63		16	36.15 ± 0.88 in HPs 38.36 ± 1.24 in POAG Up-regulated
25	Tanaka et al.,2014	Japan	Aqueous humor	20	Microarray system	10	4 cataract and POAG 4 POAG 2 PEX	72.8 (± 3)	4/6	10	5 cataract and 5 epiretinal membrane	71.1 (± 2.6)	7/3	4507	0.728 Down-regulated
26	Tanaka et al.,2014	Japan	Aqueous humor	20	Microarray system	10	4 cataract and POAG 4 POAG 2 PEX	72.8 (± 3)	4/6	10	5 cataract and 5 epiretinal membrane	71.1 (± 2.6)	7/3	3620-5p	0.622 Down-regulated
27	Tanaka et al.,2014	Japan	Aqueous humor	20	Microarray system	10	4 cataract and POAG 4 POAG 2 PEX	72.8 (± 3)	4/6	10	5 cataract and 5 epiretinal membrane	71.1 (± 2.6)	7/3	4484	1.639 Up-regulated
28	Tanaka et al.,2014	Japan	Aqueous humor	20	Microarray system	10	4 cataract and POAG 4 POAG 2 PEX	72.8 (± 3)	4/6	10	5 cataract and 5 epiretinal membrane	71.1 (± 2.6)	7/3	5001-5p	0.718 Down-regulated
29	Tanaka et al.,2014	Japan	Aqueous humor	20	Microarray system	10	4 cataract and POAG 4 POAG 2 PEX	72.8 (± 3)	4/6	10	5 cataract and 5 epiretinal membrane	71.1 (± 2.6)	7/3	6132	0.71 Down-regulated
30	Tanaka et al.,2014	Japan	Aqueous humor	20	Microarray system	10	4 cataract and POAG 4 POAG 2 PEX	72.8 (± 3)	4/6	10	5 cataract and 5 epiretinal membrane	71.1 (± 2.6)	7/3	6515-3p	1.413 Up-regulated
31	Tanaka et al.,2014	Japan	Aqueous humor	20	Microarray system	10	4 cataract and POAG 4 POAG 2 PEX	72.8 (± 3)	4/6	10	5 cataract and 5 epiretinal membrane	71.1 (± 2.6)	7/3	4467	0.79 Down-regulated
32	Tanaka et al.,2014	Japan	Aqueous humor	20	Microarray system	10	4 cataract and POAG 4 POAG 2 PEX	72.8 (± 3)	4/6	10	5 cataract and 5 epiretinal membrane	71.1 (± 2.6)	7/3	3663-3p	1.432 Up-regulated
33	Tanaka et al.,2014	Japan	Aqueous humor	20	Microarray system	10	4 cataract and POAG 4 POAG 2 PEX	72.8 (± 3)	4/6	10	5 cataract and 5 epiretinal membrane	71.1 (± 2.6)	7/3	187-5p	0.524 Down-regulated
34	Tanaka et al.,2014	Japan	Aqueous humor	20	Microarray system	10	4 cataract and POAG 4 POAG 2 PEX	72.8 (± 3)	4/6	10	5 cataract and 5 epiretinal membrane	71.1 (± 2.6)	7/3	4433-3p	1.474 Up-regulated
35	Tanaka et al.,2014	Japan	Aqueous humor	20	Microarray system	10	4 cataract and POAG 4 POAG 2 PEX	72.8 (± 3)	4/6	10	5 cataract and 5 epiretinal membrane	71.1 (± 2.6)	7/3	6717-5p	1.909 Up-regulated
36	Tanaka et al.,2014	Japan	Aqueous humor	20	Microarray system	10	4 cataract and POAG 4 POAG 2 PEX	72.8 (± 3)	4/6	10	5 cataract and 5 epiretinal membrane	71.1 (± 2.6)	7/3	6722-3p	0.517 Down-regulated
37	Tanaka et al.,2014	Japan	Aqueous humor	20	Microarray system	10	4 cataract and POAG 4 POAG 2 PEX	72.8 (± 3)	4/6	10	5 cataract and 5 epiretinal membrane	71.1 (± 2.6)	7/3	4725-3p	1.588 Up-regulated
38	Tanaka et al.,2014	Japan	Aqueous humor	20	Microarray system	10	4 cataract and POAG 4 POAG 2 PEX	72.8 (± 3)	4/6	10	5 cataract and 5 epiretinal membrane	71.1 (± 2.6)	7/3	1202	1.388 Up-regulated
39	Tanaka et al.,2014	Japan	Aqueous humor	20	Microarray system	10	4 cataract and POAG 4 POAG 2 PEX	72.8 (± 3)	4/6	10	5 cataract and 5 epiretinal membrane	71.1 (± 2.6)	7/3	3197	1.454 Up-regulated
40	Tanaka et al.,2014	Japan	Aqueous humor	20	Microarray system	10	4 cataract and POAG 4 POAG 2 PEX	72.8 (± 3)	4/6	10	5 cataract and 5 epiretinal membrane	71.1 (± 2.6)	7/3	4749-5p	0.794 Down-regulated
41	Tanaka et al.,2014	Japan	Aqueous humor	20	Microarray system	10	4 cataract and POAG 4 POAG 2 PEX	72.8 (± 3)	4/6	10	5 cataract and 5 epiretinal membrane	71.1 (± 2.6)	7/3	1260b	0.659 Down-regulated
42	Tanaka et al.,2014	Japan	Aqueous humor	20	Microarray system	10	4 cataract and POAG 4 POAG 2 PEX	72.8 (± 3)	4/6	10	5 cataract and 5 epiretinal membrane	71.1 (± 2.6)	7/3	4634	0.738 Down-regulated
43	Tanaka et al.,2014	Japan		20	Microarray system	10	4 cataract and POAG 4 POAG 2 PEX	72.8 (± 3)	4/6	10	5 cataract and 5 epiretinal membrane	71.1 (± 2.6)	7/3	4259	1.981 Down-regulated
44	Tanaka et al.,2014	Japan	Aqueous humor	20	Microarray system	10	4 cataract and POAG 4 POAG 2 PEX	72.8 (± 3)	4/6	10	5 cataract and 5 epiretinal membrane	71.1 (± 2.6)	7/3	92a-2-5p	2.331 Up-regulated
45	Tanaka et al.,2014	Japan	Aqueous humor	20	Microarray system	10	4 cataract and POAG 4 POAG 2 PEX	72.8 (± 3)	4/6	10	5 cataract and 5 epiretinal membrane	71.1 (± 2.6)	7/3	4449	1.926 Up-regulated
46	Tanaka et al.,2014	Japan	Aqueous humor	20	Microarray system	10	4 cataract and POAG 4 POAG 2 PEX	72.8 (± 3)	4/6	10	5 cataract and 5 epiretinal membrane	71.1 (± 2.6)	7/3	1587	0.483 Down-regulated
47	Tanaka et al.,2014	Japan	Aqueous humor	20	Microarray system	10	4 cataract and POAG 4 POAG 2 PEX	72.8 (± 3)	4/6	10	5 cataract and 5 epiretinal membrane	71.1 (± 2.6)	7/3	486-3p	0.436 Down-regulated
48	Tanaka et al.,2014	Japan	Aqueous humor	20	Microarray system	10	4 cataract and POAG 4 POAG 2 PEX	72.8 (± 3)	4/6	10	5 cataract and 5 epiretinal membrane	71.1 (± 2.6)	7/3	3185	0.642 Down-regulated
49	Tanaka et al.,2014	Japan	Aqueous humor	20	Microarray system	10	4 cataract and POAG 4 POAG 2 PEX	72.8 (± 3)	4/6	10	5 cataract and 5 epiretinal membrane	71.1 (± 2.6)	7/3	940	0.303 Down-regulated
50	Tanaka et al.,2014	Japan	Aqueous humor	20	Microarray system	10	4 cataract and POAG 4 POAG 2 PEX	72.8 (± 3)	4/6	10	5 cataract and 5 epiretinal membrane	71.1 (± 2.6)	7/3	3652	0.335 Down-regulated
51	Tanaka et al.,2014	Japan	Aqueous humor	20	Microarray system	10	4 cataract and POAG 4 POAG 2 PEX	72.8 (± 3)	4/6	10	5 cataract and 5 epiretinal membrane	71.1 (± 2.6)	7/3	3135b	0.626 Down-regulated
52	Tanaka et al.,2014	Japan	Aqueous humor	20	Microarray system	10	4 cataract and POAG 4 POAG 2 PEX	72.8 (± 3)	4/6	10	5 cataract and 5 epiretinal membrane	71.1 (± 2.6)	7/3	5572	0.437 Down-regulated
53	Tanaka et al.,2014	Japan	Aqueous humor	20	Microarray system	10	4 cataract and POAG 4 POAG 2 PEX	72.8 (± 3)	4/6	10	5 cataract and 5 epiretinal membrane	71.1 (± 2.6)	7/3	4640-5p	0.525 Down-regulated

*NO* number, *SD* standard deviation, *F* female, *M* male, *RT-PCR* real-time polymerase chain reaction, *POAG* primary open angle glaucoma, *PEX* pseudoexfoliation, *OHT* ocular hypertension

### Differentially expressed microRNAs

After the screening of the 321 articles, finally, 6 of them were included [15–20]. Fifty-two differentially expressed mature microRNAs in these selected studies compared 115 glaucoma patients with 108 controls. The total number of individual microRNAs found in the included studies was 52, of which 24 were down-regulated, and 28 were up-regulated. The diagnostic accuracy of miR-210-3 was measured in two different scenarios: a screening set with a smaller sample size, followed by a validation in a larger group of patients; it was up-regulated in both steps [17]. Of the 52 individual microRNAs, 3, 3, 11, and 36 were taken from serum, plasma, tears, and aqueous humor, respectively.

All the patients' plasma and serum microRNA samples were up-regulated [15, 17]. However, most of the patients' tear samples contained microRNAs that were up-regulated [18, 19] compared with the control group (9 versus 2). In contrast, of the 36 samples of aqueous humor microRNAs, 22 were down-regulated and 14 were up-regulated [16, 20].

### Meta-analysis of diagnostic test accuracy studies

**Fig. 4:**
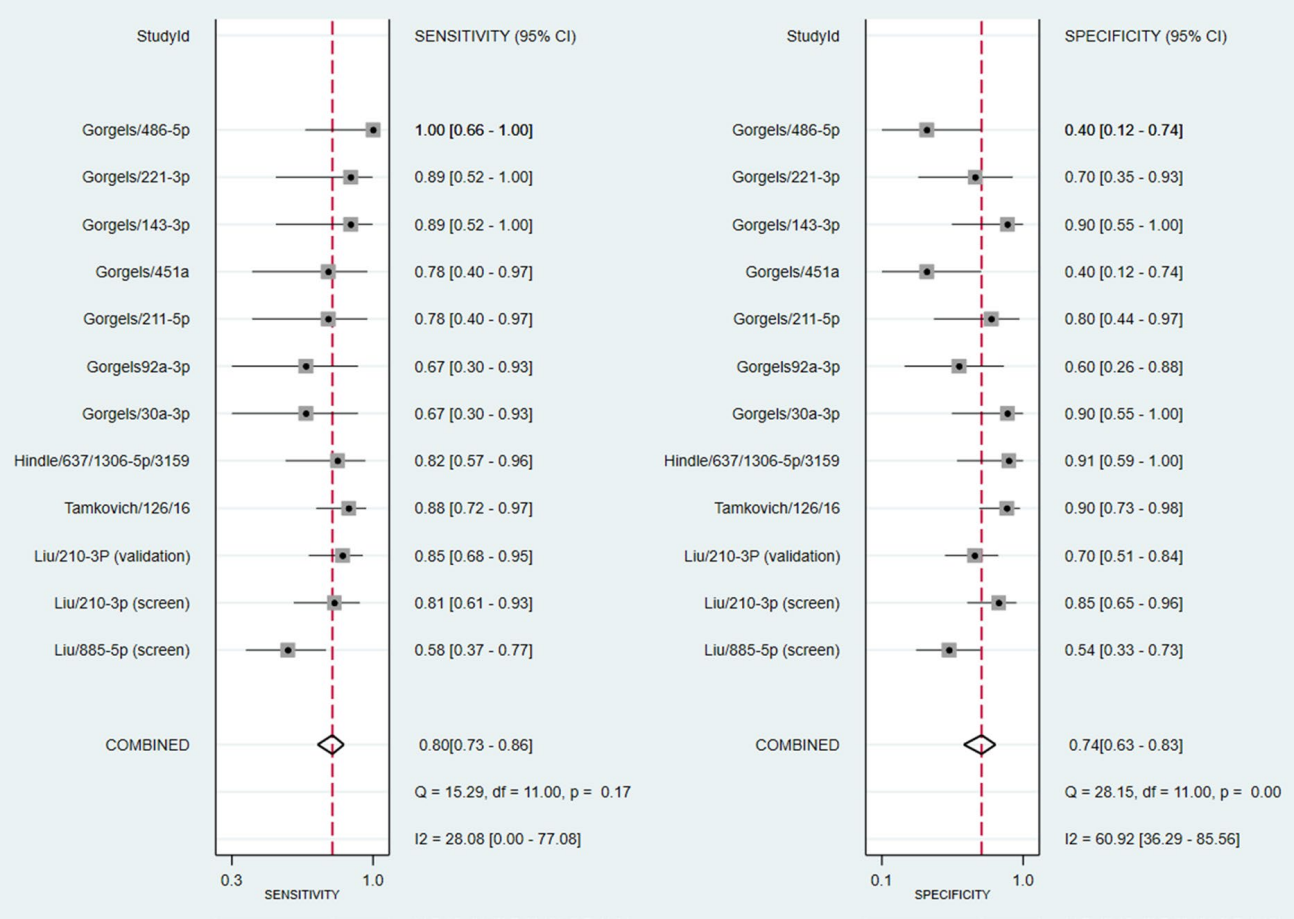
Forest plots and meta‑analyses of studies showing the pooled sensitivity and specificity of circulating microRNAs for diagnosing glaucoma patients

**Fig. 5:**
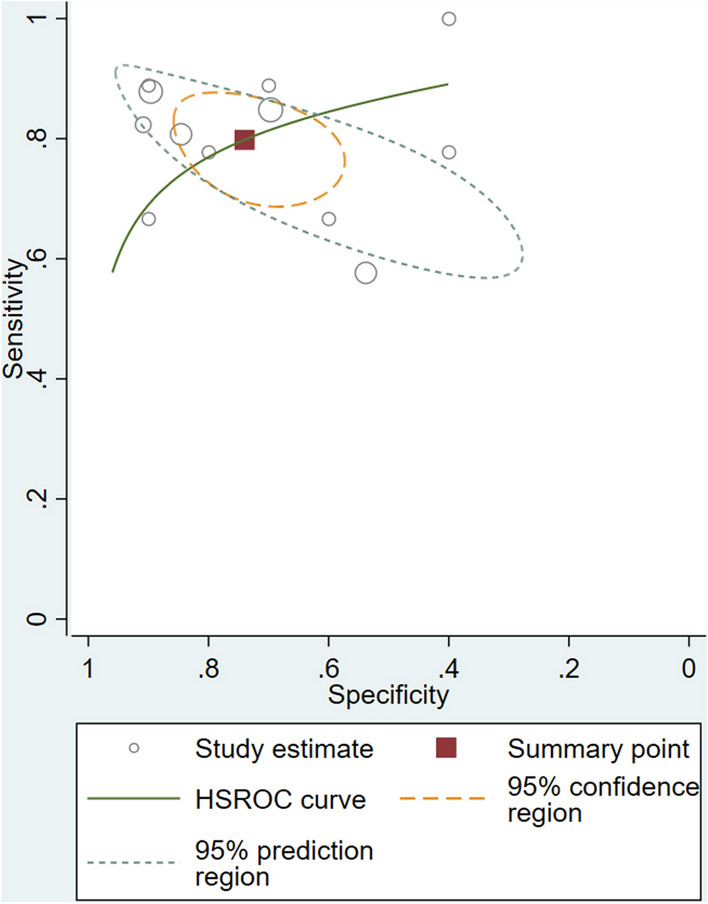
Summary of AUROC of circulating microRNAs for the diagnosis of glaucoma patients; white circles represent single study data while the red square represents the sensitivity/specificity pair at the summary operating point

**Fig. 6:**
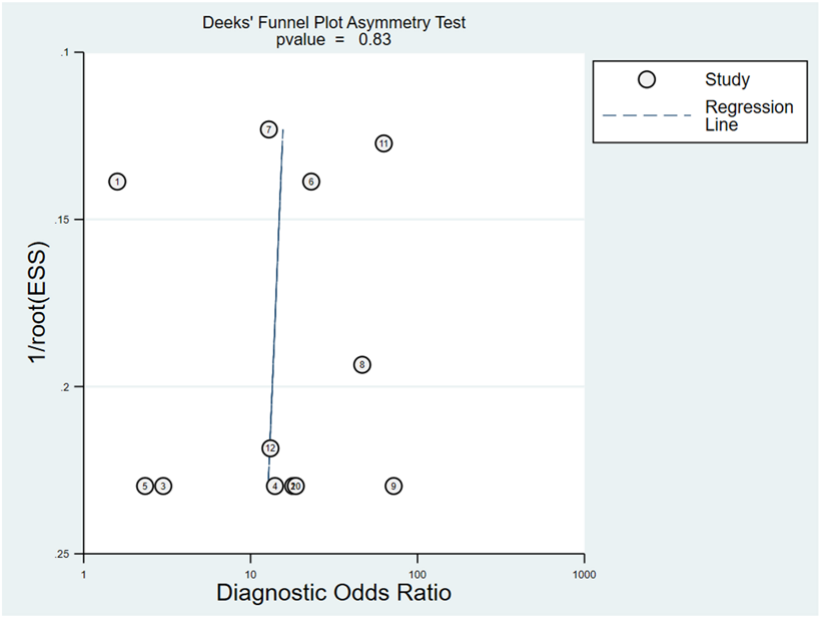
Deeks’ funnel plots were used to estimate publication bias for discrimination of microRNAs in patients with glaucoma

The meta-analysis included studies that detected a single dysregulated microRNA in glaucoma cases compared to controls and the corresponding AUC, sensitivity, and specificity for the discovered microRNA. Using these criteria, four studies identified 12 individual microRNAs. The associated forest plot depicts the sensitivity (95% CIs) and specificity (95% CIs) for each microRNA (Fig. 4). Overall sensitivity and specificity of the 12 individual microRNAs in the diagnosis of glaucoma were 80% [95% CI 73–86%, test of heterogeneity: Q = 15.29, I^2^ = 28.08%, *p-value* = 0.17] and 74% [95% CI 63–83%, test of heterogeneity: Q = 28.15, I^2^ = 60.9%, *p-value* = 0.00], respectively. These findings suggest that utilizing microRNAs as biomarkers has a high discriminative ability in detecting glaucoma. For glaucoma detection, the slope coefficient was related to a *p-value* of 0.83 (Fig. 5), indicating no publication bias in our meta-analysis. In addition, the pooled diagnostic odds ratio (DOR) of the 12 individual microRNAs in the diagnosis of glaucoma was 11.2 [95% CI 5.3–24]. Afterward, random-effects models were used to re-analyze the data, and the diagnostic threshold was investigated. The Spearman's correlation coefficient was − 0.130 (*p-value* = 0.687), indicating that the diagnostic threshold was not responsible for the heterogeneity. Disparities in study techniques, specimen type, endogenous reference, or total sample size may also contribute to the existing heterogeneity. The publication bias of meta-analysis in diagnostic accuracy was investigated using Deeks' funnel plot asymmetry test, depicted in Fig. 6. The most well-known reporting bias is publication bias. It is the outcome of relevant trials being published or not, depending on the type and direction of the results. A paper, for instance, is more likely to be published if the findings are significant. Following subgroup analysis, it has been apparent that the most sensitive microRNA was mir-486-5p from aqueous humor (100%, 95% CI 66–100%). The most specific microRNAs were sampled from the plasma (mir-637, mir-1306-5p, and mir-3159). The data for subgroup analysis are provided in Additional file 1.

### Network analysis

#### microRNA target identification and PPI network construction

The microRNA targets were found using the MirTarBase and were used in the STRING database to construct the final PPI network. The final network had 74 different genes with 120 interactions between them.

#### Identification of hub genes

**Fig. 7:**
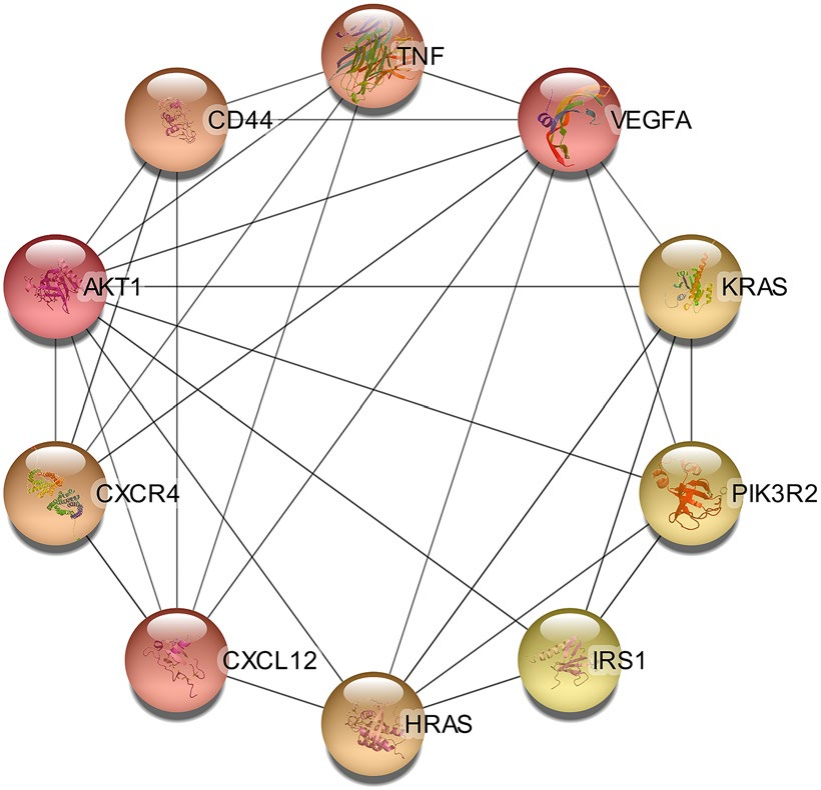
The top ten genes are based on the MCC score

Genes were sorted according to their maximal clique centrality (MCC) values, and the top ten genes with the highest MCC value are depicted in Fig. 7. Vascular endothelial growth factor A (VEGF-A) plays a vital role in angiogenesis regulation in normal and abnormal states, such as tumor angiogenesis [21]; AKT1 is a member of the serine/threonine AGC protein kinase family and is associated with cellular growth, metabolism, survival, and proliferation [22]; CXCL12 participates in a series of processes, such as inflammation and leukocyte trafficking, regulation of cell viability, and extracellular matrix remodeling [23]. The HRAS gene is involved primarily in regulating cell division [24].

#### Network communities

**Fig. 8:**
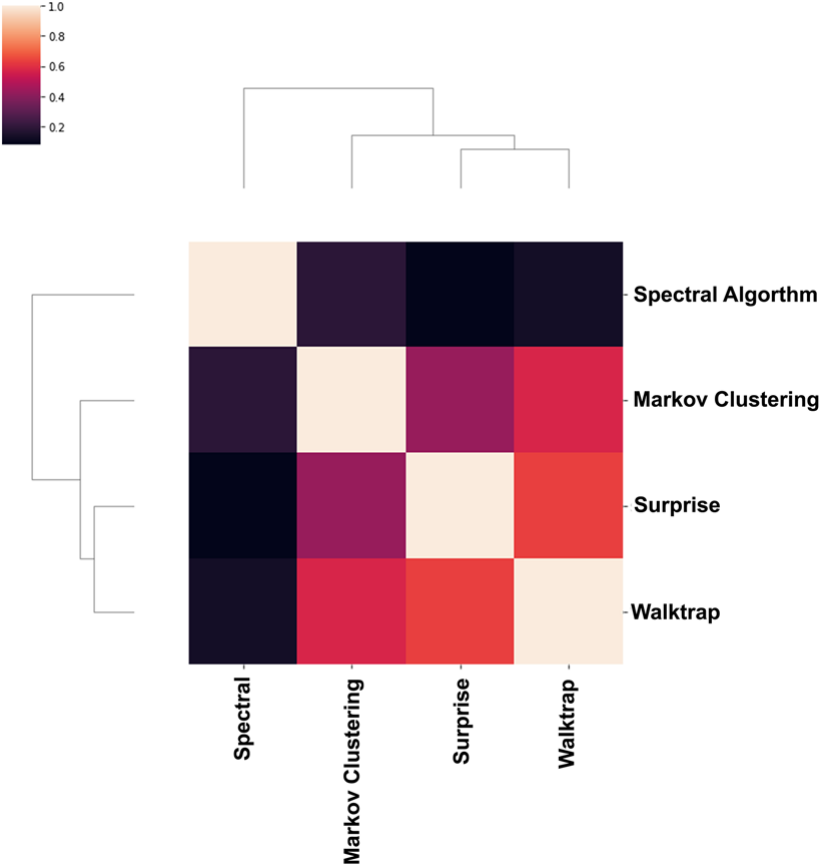
The correlation matrix between four algorithms in the benchmark (the darker the color, the lower the correlation, and vice versa)

**Fig. 9:**
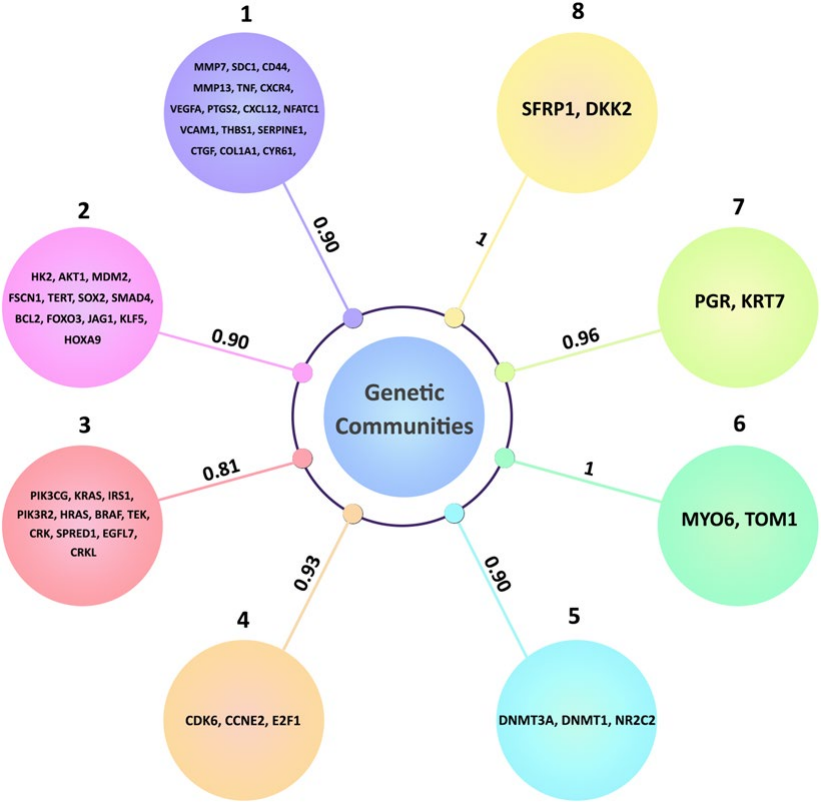
Most significant genetic communities in glaucoma with their corresponding Crank score

In the benchmark of the four different algorithms for community detection, walk-trap, a widely used algorithm in graph partitioning, achieved the first rank and was applied to the network to find the most important communities. Walk-trap is a hierarchical clustering algorithm based on a random walk iteration, which means members of a community have an overall shorter distance random walk than the rest of the network [25]. Figure 8 shows the community correlation matrix of the mentioned algorithms. The walk-trap results are consistent with the surprise and Markov clustering algorithms, but the spectral algorithm has found a different set of communities. The convergence of walk-trap, surprise, and Markov clustering algorithm communities may potentiate the probability of finding the actual pathobiological processes in glaucoma. The community prioritization ranking for the walk-trap result is provided in Table 2. In total, eight non-overlapping communities are found in the network (Fig. 9). The biggest community has 16 genes, and the smallest has only two. The details of each community, besides each community's gene ontology and biological processes, are provided in Table 2.

**Table 2:** The community ranking score, community components, and Gene Ontology (GO) of each community

Community	Crank score	Community members	Gene Ontology biological process
Cmt1	0.909787	MMP7, SDC1, CD44, MMP13, TNF, CXCR4, VEGFA, PTGS2, CXCL12, VCAM1, THBS1, SERPINE1, CTGF, COL1A1, CYR61, NFATC1	Extracellular structure organization
Cmt2	0.909787	HK2, AKT1, MDM2, FSCN1, TERT, SOX2, SMAD4, BCL2, FOXO3, JAG1, KLF5, HOXA9	Negative regulation of extrinsic Apoptotic signaling pathway in the absence of ligand
Cmt3	0.811173	PIK3CG, KRAS, IRS1, PIK3R2, HRAS, BRAF, TEK, CRK, SPRED1, EGFL7, CRKL	MAPK cascade
Cmt4	0.938829	CDK6, CCNE2, E2F1	Cell cycle g1/s phase transition
Cmt5	0.909787	DNMT3A, DNMT1, NR2C2	DNA alkylation
Cmt6	1	MYO6, TOM1	Protein transport
Cmt7	0.962558	PGR, KRT7	Steroid hormone-mediated Signaling pathway
Cmt8	1	SFRP1, DKK2	Regulation of canonical wnt signaling pathway

Interestingly, after using the CRANK algorithm on these communities, we could show that two small communities could get a higher rank, indicating the importance of community ranking in network analyses. Intriguingly, the eighth community, which contains SFRP1 and DKK2 genes, is involved in the WNT signaling pathway, and recent research findings support the crucial role of this pathway in regulating intraocular pressure [26, 27]. The other simultaneously first-ranked community is the sixth community, which involves the MYO6 and TOM1 genes. The TOM1 gene is involved in vesicular trafficking at the endosome, and mutations in this gene may lead to a combination of brain–eye–muscle anomalies [28]. On the other hand, Opineurin's weakened interaction with these two proteins results in dysregulated autophagosome maturation and clearance of inclusion bodies, eventually leading to glaucoma [29].

## Discussion

MicroRNAs are one of the major epigenetic drivers in glaucoma. To the best of our knowledge, this paper is the first attempt to conduct a systematic review, meta-analysis, and genetic network analysis of microRNA profiling in glaucoma patients. The regulatory effects of microRNAs on their target genes may demonstrate some clues to the pathogenesis of glaucoma. Discovering diagnostic microRNAs that meticulously correlate with the presence of the disease could be beneficial in the early diagnosis and screening of glaucoma patients.

The connection between microRNAs and aqueous humor production and absorption, trabecular meshwork, the apoptosis of retinal ganglion cells, and glaucoma have been investigated extensively in human and other animals' eyes, like rats [15, 30–32]. It is worth mentioning that the expression patterns of the microRNAs in glaucoma are determined by the disease type and the clinical course of the disease.

The two most frequently dysregulated microRNAs in all studies include miR-486-5p and miR-143-3p. In this study, microRNAs altered in at least two studies were validated to find the corresponding target genes responsible for glaucoma development. AKT1, VEGF-A, CXCL12, and HRAS were the most notable targets.

MiR-486-5p is a potential biomarker for prognosis, diagnosis, and therapeutic target in several cancers [33]. Also, it has been reported to be correlated with disease severity and inflammation in sepsis [34]. Significant downregulation of miR-486-5P compared with the control group was indicated in two studies based on aqueous humor and plasma [16, 35].

On the other hand, it has been demonstrated that MiR-143-3p plays a tumor suppressor role in several tumors [36]; researchers identified the upregulation of this biomarker in patients with glaucoma [16].

Regarding hub genes in the network, except for VEGF-A, little is known about the exact function of the remaining genes. In [37], aqueous humor concentrations of VEGF-A had significantly higher levels in the eyes of patients with neovascular glaucoma. Correspondingly, there is evidence that patients with glaucoma might benefit from anti-VEGF-A therapy [38, 39]. Intriguingly, the results of this work have shed new light on the possible remarkable roles of AKT1, CXCL12, and HRAS genes in the pathogenesis of glaucoma.

Gene community findings are in line with those found in the literature, where the increased IOP causes perforation, deformation, and remodeling of the lamina cribrosa (a porous layer that allows nerve fibers to pass through the eye) and eventually interruption of retrograde transport of essential trophic factors to the retinal ganglion cells [40]; Previous studies indicate that perturbations in axonal transport are one of the earliest findings in the pathogenesis of glaucoma. A recent review of studies undertaken looking at the role of axonal transport in glaucoma has shown that both antegrade and retrograde protein transport are severely disrupted in glaucoma [41]. A causal loop exists as we do not exactly know whether the derangement in axonal transport causes increased IOP or vice versa. As indicated in our study, MYO6 is an unconventional reverse-direction myosin that is required for the degradation of harmful cellular components and the structural integrity of the Golgi apparatus via the p53-dependent pro-survival pathway [42].

Trabecular meshwork (TM) is a fenestrated structure that permits the aqueous humor to be drained from the eye [43]. The production rate of aqueous humor is monotonous; thus, the regulation of IOP is managed by controlling the outflow rate [44]. The increased IOP leads to the extracellular remodeling matrix (ECM) by activating a group of matrix metalloproteinases (MMPs) that instigate outflow rates by opening the pores. The types and expression levels of MMPs vary by the disease; for example, in age-related macular degeneration, the expression of MMP-2 and MMP-9 is significantly reduced [45]. Here we showed that MMP-7 (a matrilysin) and MMP-13 (also known as collagenase-3) are the most important MMPs in glaucoma. It should be mentioned that MMP-7, which is one of the smallest human MMPs, hypothetically can play a dual role in glaucoma; it facilitates ECM remodeling to increase outflow from the anterior chamber; on the other hand, it can cleave cell-surface molecules such as Fas-ligand and pro-TNF-α that induce apoptosis [46].

There is strong evidence for the presence of the canonical WNT signaling pathway in TM. sFRP1, a WNT signaling inhibitor, is significantly expressed in TM and regulated by microRNAs, not DNA methylation. Increased expression of sFRP1 is associated with TM stiffness and subsequently reduced outflow, loss of cell–cell communication, and ultimately apoptosis [47].

Steroid use is one of the risk factors for the pathogenesis of glaucoma [4]. As mentioned in Table 2, PGR and KRT7 in the steroid hormone-mediated signaling pathway are the most affected genes. Previous studies do not provide direct evidence of these genes in steroid-induced glaucoma; hence, further experiments are expected to improve the understanding of steroid-induced glaucoma concerning these two genes. Moreover, according to the literature, corticosteroids can inhibit MMPs and subsequently reduce the adaptation of the ECM to increased pressure [48].

A potential issue we need to consider is the variability of the methods for assessing microRNA profiling, which might compromise their reliability and reproducibility. One of the limitations of the current study is that the microRNAs' sources are different, which could affect the subsequent analyses. MicroRNA releasing mechanisms might also be tissue-specific. Furthermore, it would also be interesting to consider TM regions where the biopsy has been taken because studies have shown that the rate of drainage can be different in TM (high-flow vs. low-flow draining parts) [49]. Moreover, our study findings are solely based on a meta-analysis, and the results might also be influenced by the small sample size, control heterogeneity in case–control studies, or the ethnicity of the glaucoma patients. Future large cohort studies are needed to thoroughly assess the clinical relevance of the obtained results. Despite these limitations, the current study still tries to provide a representative overview of the microRNAs and their target genes involved in the pathogenesis of glaucoma for directing future experiments, providing novel drug targets, and eventually cutting down the immense burden of this sight-threatening disease.

## Conclusion

Glaucoma is a multifactorial sight-threatening disease with the complex interaction of genetic and environmental components. Emerging research reveals epigenetics as a key element in developing and preventing the disease. Epigenetic effects are conveyed through pathways regulating apoptosis, ECM protein synthesis, and inflammation. These pathways do not bear equal weight. Using the network analysis, we can estimate the extent of their influences.

## Supplementary Information

**Additional file 1: Figure S1.** The Hierarchical Summary Receiver Operating Characteristic (HSROC) curve of circulating miRNAs for the diagnosis glaucoma patients. **Figure 2.** The scatter plot of the Likelihood ratio of circulating miRNAs for the diagnosis glaucoma patients. **Figure S3.** Fagan's plot for pre-test and post-probabilities with likelihood ratios. **Figure S4.** Probability modifying plot. **Figure S5.** Subgroup analysis of the microRNAs based on the sample type. **Figure 6.** Sensitivity vs specificity plot for various microRNAs based on sample site subgroups.

## Data Availability

The datasets used and/or analyzed during the current study are available from the corresponding author on reasonable request.
